# Comparative Analysis of Tat-Dependent and Tat-Deficient Natural Lentiviruses

**DOI:** 10.3390/vetsci2040293

**Published:** 2015-09-29

**Authors:** Deepanwita Bose, Jean Gagnon, Yahia Chebloune

**Affiliations:** Pathogénèse et Vaccination Lentivirales, PAVAL Lab., Université Joseph Fourier Grenoble 1, Bat. NanoBio2, 570 rue de la Chimie, BP 53, 38041, Grenoble Cedex 9, France; E-Mails: deepanwita.bose@ujf-grenoble.fr (D.B.); jean.gagnon@ujf-grenoble.fr (J.G.)

**Keywords:** Lentiviruses, HIV, CAEV, pathogenesis, Tat, latency

## Abstract

The emergence of human immunodeficiency virus (HIV) causing acquired immunodeficiency syndrome (AIDS) in infected humans has resulted in a global pandemic that has killed millions. HIV-1 and HIV-2 belong to the lentivirus genus of the *Retroviridae* family. This genus also includes viruses that infect other vertebrate animals, among them caprine arthritis-encephalitis virus (CAEV) and Maedi-Visna virus (MVV), the prototypes of a heterogeneous group of viruses known as small ruminant lentiviruses (SRLVs), affecting both goat and sheep worldwide. Despite their long host-SRLV natural history, SRLVs were never found to be responsible for immunodeficiency in contrast to primate lentiviruses. SRLVs only replicate productively in monocytes/macrophages in infected animals but not in CD4+ T cells. The focus of this review is to examine and compare the biological and pathological properties of SRLVs as prototypic Tat-independent lentiviruses with HIV-1 as prototypic Tat-dependent lentiviruses. Results from this analysis will help to improve the understanding of why and how these two prototypic lentiviruses evolved in opposite directions in term of virulence and pathogenicity. Results may also help develop new strategies based on the attenuation of SRLVs to control the highly pathogenic HIV-1 in humans.

## 1. Background

HIV type 1 and 2, like small ruminant lentiviruses SRLVs, belong to the lentivirus genus of the *Retroviridae* family. They are small single-stranded enveloped RNA viruses characterized by reverse transcription of their RNA into double-stranded DNA for their replication. Additional members of this genus are the simian immunodeficiency viruses (SIVs) which infect various species of monkeys, bovine immunodeficiency virus (BIV) which infects cattle, feline immunodeficiency (FIV) which infects domestic cats and a variety of wild felids, equine infectious anemia virus (EIAV) which infects horses, and the small ruminant lentiviruses (SRLVs) with the prototypic caprine arthritis encephalitis virus (CAEV) and Maedi Visna Virus (MVV) which infect mainly goats and sheep, respectively. It has been well established now that HIV-1 and HIV-2 arose in humans following recent zoonosis of SIVcpz from chimpanzees (*Pan troglodyte*), SIVgor from gorillas, and SIVsmm from Sooty mangabey macaques, respectively [1,2,3]. The viruses have repeatedly crossed the species barrier, caused infections, and adapted into human cells to become highly replication-competent and transmissible from human to human, resulting in increased pathogenesis in their new host. 

Since the discovery of HIV-1 by Montagnier and Barré-Sinoussi’s laboratory [4] over three decades ago, there have been nearly 80 million individuals infected and, prior to the development of efficient therapy, more than 35 million worldwide have progressed to AIDS and died from disease. Today there are over 35 million individuals living infected with HIV-1.

AIDS is characterized by low CD4+ T cell counts (below 200 cells/µL), hemogram abnormalities, chronic immune activation, and the occurrence of opportunistic infections. AIDS is often preceded by the co-receptor usage shift of the virus from CCR5 (macrophage-tropic) expressed predominantly at the surface of memory CD4+ T cells to CXCR4 (lymphocyte-tropic) at the surface of activated naïve CD4+ T cells, leading to severe depletion of these cells and impaired regeneration of the immune system [5]. The molecular mechanisms responsible for the CCR5 to CXCR4 switch remain still to be determined. 

**Figure 1:**
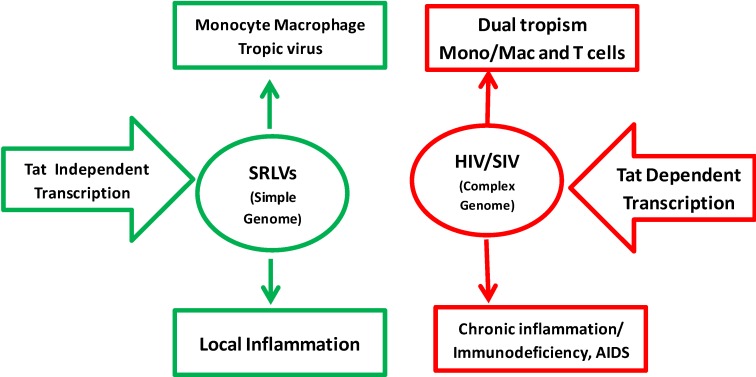
Comparison of SRLV and HIV-1 properties. SRLV replication is restricted to monocyte/macrophage cell lineage. HIV-1 can target both monocytes/macrophages and CD4+ T cells using CD4 as the main receptor and either CCR5 or CXCR4 as the co-receptor. The replication of HIV/SIV is highly dependent on Tat transactivation, while replication of SRLVs is fully independent and constitutive. The pathogenesis of SRLVs is characterized by local inflammatory diseases in target organs while HIV-1 induces chronic systemic inflammation and immunodeficiency leading to AIDS and multi-organ degenerative diseases.

MVV causing disease was intensely studied in Icelandic sheep following the import of Karakul Asian breed sheep from Germany in 1933 to genetically enrich local breeds. The sheep were quarantined and distributed to different places in Iceland. Within a span of few years, Icelandic sheep started showing signs of diseases, mostly lung (maedi) and CNS (visna) diseases [6,7]. CAEV causing disease was identified in the 1970s when caprine flocks showed neurological symptoms in kids and synovial arthritis in adults. In cell culture, MVV shows markedly more cytopathogenicity in infected monolayer cells than CAEV. The major tropism of MVV and CAEV is for macrophages and dendritic cells but not CD4+ T cells *in vivo* [8,9]. Although HIV-1 also infects these cells, the virus replication occurs predominantly in CD4+ T lymphocytes in infected humans. Thus, SRLVs only cause productive infection, inflammation, and chronic degenerative diseases in 20–50% of naturally infected animals; this affects the central nervous system, lungs, udder, and joints. In contrast, HIV-1 infection in humans is associated with impairment of the immune system in nearly 100% of infected individuals. In the absence of therapy, infected patients undergo progressive destruction of CD4+ T lymphocytes, leading to AIDS and death due to opportunistic infections (Figure 1).

Here we will examine and compare the biological, pathological, and host/pathogen interactions in SRLVs of sheep and goats *versus* HIV-1/SIV of human and non-human primates. 

## 2. Genome Organization of SRLVs and HIV-1 

**Figure 2:**
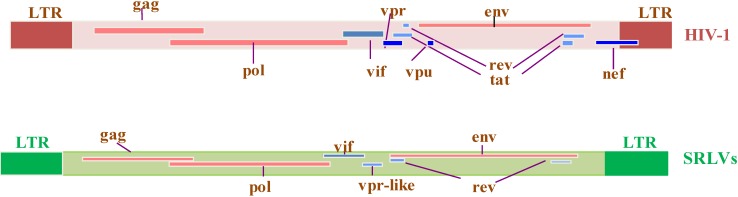
Genomic organization of HIV-1 and SRLV proviruses. Both genomes have the structural and enzyme *gag*, *pol*, and *env* genes (solid red bars), with LTRs at the 5’ and 3’ends (solid brown and green rectangles in HIV-1 and SRLVs, respectively) common to all retroviruses. The six additional open reading frames (*vif*, *vpr*, *vpu*, *tat*, *rev*, and *nef*) that encode regulatory and accessory proteins are indicated (solid blue bars), while the SRLV genomes have only three additional open reading frames (*vif*, *vpr-like*, and *rev*) [13].

The HIV-1 genome contains the classical *gag*, *pol*, and *env* genes found in all replication-competent retroviruses. The genome has also three regulatory genes, *tat*, *rev* and *vif*, whose products are necessary for efficient replication, and three auxiliary genes, *nef*, *vpr* and *vpu*, whose products are involved in virus/host interactions and pathogenesis. The HIV-1 genome with its nine major genes is more complex than SRLV genomes which, in addition to *gag*, *pol* and *env*, contain only two regulatory *vif* and *rev* genes and one auxiliary gene called “*tat*”. The latter from here on will be referred to as *vpr*-like gene [10]*.*The low pathogenicity of SRLVs compared to HIV-1 can potentially be linked to the absence of proteins encoded by *nef*, *vpu*, *and tat* genes (Figure 2).

The HIV-1 *gag* gene produces a 55 kilodalton (kD) Gag precursor protein (Gag Pr-55) that is subsequently cleaved to generate the matrix p17, the capsid p24, the nucleocapsid p7 proteins, and the p6, which is critical for virus budding. The 160 kD Env glycoprotein precursor (gp160) is expressed from singly spliced viral mRNA and then is matured by cellular protease cleavage to generate the surface gp120 and transmembrane gp41 mature Env glycoproteins. The catalytic proteins are produced from a precursor protein encoded by the *pol* gene which, following cleavage, generates the protease (Pro p10), reverse transcriptase (RT p50), and integrase (IN p31). Proteases are known to play essential roles in many biological processes. They catalyze the hydrolysis of peptide bonds with high sequence selectivity and catalytic proficiency. HIV-1 protease, a member of the aspartic protease family, is a symmetrically assembled homodimer consisting of two identical subunits of 99 amino acids. Both subunits are involved in the catalytic activity (through an aspartic acid at codon 25). It is responsible for the cleavage of Gag-Pol, where Gag and Pol can be released during budding. In the absence of functional protease the viral assembly is not impaired, but the resulting particles are non-infectious [11,12]. RT has two enzymatic activities, a DNA polymerase that can copy either a DNA or a RNA template, and a RNase H activity that removes RNA from the RNA/DNA intermediate. Two different domains of RT are implicated in these two activities and they cooperate to convert the genomic RNA into a double-stranded linear DNA. The resulting double-stranded viral DNA is then transported into the nucleus and integrated in the host genome by IN [14].

### 2.1. Virus-Encoded Regulatory Proteins

#### 2.1.1. Regulatory Tat 

Tat is a key protein that regulates positively the expression of all genome encoded proteins in some of the lentiviruses. Detailed description of Tat and Tat functions are discussed in Section 2.3 and Section 2.4.

#### 2.1.2. Regulatory Rev

Rev and Tat are expressed early during the initial phases of the replication cycle from multi-spliced RNA templates [15,16]. HIV-1 Rev is a 116-amino-acid phosphoprotein translated from fully spliced viral mRNA and encodes a protein that has four functional motifs. They include (i) a nuclear localization signal (NLS) directly interacting with importin-β for the import of Rev to the nucleus; (ii) a RNA-binding domain (RBD) that specifically directs the interaction of Rev with the Rev response elements (RRE) sequence; (iii) sequences that mediate self-interactions of Rev-Rev ending with a complex formation with the RRE; and (iv) a C-terminal leucine-rich nuclear export signal (NES) that binds to the chromosomal region maintenance 1 (CRM1) for the nuclear export of unspliced and unispliced RNA [17,18,19,20].

#### 2.1.3. Regulatory Vif

Viral infectivity factor (Vif) is a 23 kD basic protein that is expressed in a Rev-dependent manner, largely localized in the cytoplasm of infected cells, and is essential for HIV-1 replication in lymphocytes and macrophages. Many studies using Δ*vif* mutant genomes have demonstrated that these genotypes produce virions that are non-infections, yet there is no difference in the protein or RNA contents of Δ*vif* compared to the wild type [21,22,23]. The main role of Vif is to suppress the host antiviral defense mechanism involving the DNA editing enzyme APOBEC3G. Studies have demonstrated that Vif neutralizes the potent intracellular defense pathway of APOBEC3G that protects host cells from a retrovirus. Several members of the APOBEC family act on single-stranded DNA or RNA and they alter the nucleotide sequence through cytidine deamination, converting cytidine to uridine and thus providing an intrinsic immunity to the host [24], which is an edit-dependent process [25,26]. APOBEC inhibits the retrovirus by several mechanisms which are edit-independent processes as well [27]. 

### 2.2. Accessory Virus Gene Encoded Proteins 

#### 2.2.1 Accessory Nef 

**Figure 3:**
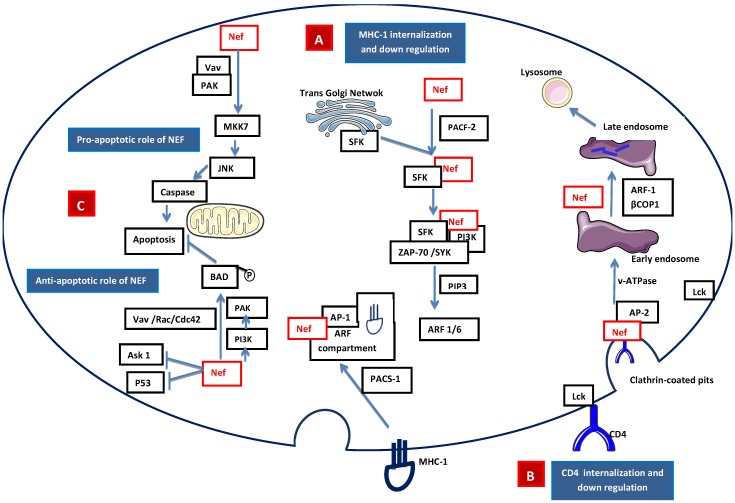
Signaling model of Nef-mediated (**A)** apoptosis/anti-apoptosis, **(B)** down-regulation of expression of MHC class I molecules, and (**C)** down-regulation of the expression of CD4 molecules. **A. Internalization of MHC-I:** Nef accelerates the endocytosis of MHC class I molecules through PI3K-dependent activation of ADP ribosylation factor 6 (ARF6)-mediated endocytosis. First, the Nef binds PACS-2 and targets to the late Golgi/TGN; at the TGN, Nef binds and activates a TGN-localized SFK. The activated Nef–SFK complex, which leads to the signal transduction pathway recruiting and activating ZAP-70/Syk, then binds to a class I PI3K. The activated PI3K causes the accumulation of phosphatidylinositol-3,4,5-triphosphate (PtdInsP_3_) (PIP3) on the inner leaflet of the plasma membrane. PIP3 recruits the ARF6 guanosine exchange factor (ARF6-GEF) to the plasma membrane. Nef mediates the internalization of MHC-I from the plasma membrane to an ARF-6 endosomal compartment. The endocytosed MHC-I forms a complex with Nef and AP-1 *via* phosphofurin acidic cluster sorting protein 1 (PACS1). **B. Internalization of CD4:** Lck disassociates from the cytoplasmic tail of CD4 and Nef is attached to the cytoplasmic tail of CD4 within clathrin-coated pits through the interaction of adaptor protein 2 (AP-2) and vacuolar ATPase (v-ATPase). This leads to the internalization of CD4 into the early endosome. During the formation of the late endosome, Nef intacts with βCOP1 and through ARF-1, which targets CD4 to the lysosomal degradation. **C. Apoptotic role of Nef:** Intrinsic Nef signals localized on plasma membrane activate MAP kinase kinase 7 (MKK7) and c-Jun N-terminal kinase (JNK) to induce caspase-dependent apoptosis, while the anti-apoptotic pathway is mediated by Fas cell surface death receptor (FAS) and tumor-necrosis factor receptor (TNFR) by inhibiting the apoptosis signal-regulating kinase (ASK1) and P53. Nef-mediated p21-activated kinase (PAK) activation involves PI3K, which acts upstream of PAK. Nef-associated PI3K and PAK phosphorylate the pro-apoptotic Bad protein to inactivate Bad, thus blocking the apoptosis mediated by BCL-2.

HIV-1 Nef is a 27 kD myristoylated protein that was initially thought to be a negative factor for virus replication and presents only in the intracellular compartment of infected cells. Later, Nef was found to have a positive effect on viral replication [28]. During HIV-1 infection, Nef down-regulates the surface expression of the CD4 and selective MHC class I receptors, thereby avoiding lytic HIV-1 superinfections and early elimination of HIV-1-infected cells by natural killer cells and cytotoxic T cells [29]. Nef induces internalization of MHC-I molecules that accumulate in the endosomal vesicles and are subsequently degraded. Nef also induces relocalization of internalized MHC-I molecules from the cell surface to the transgolgi network (TGN) [30]. MHC-I down-regulation may involve clathrin adapter protein complex 1 (AP-1) or Src family kinase-ZAP70/Syk-PI3K cascade recruited by phosphofurin acidic cluster sorting protein 1 (PACS2) [31,32,33] (Figure 3A). Nef interacts with several downstream cascades and complexes, including the adaptor complex of clathrin-coated pits and the beta subunits of COP-I coatomer, to down-regulate CD4 [34]. The CD4-p56lck complex is disrupted by Nef, allowing for the internalization of CD4 and the diversion of internalized CD4 to the lysosomal pathway which results in its destruction through the phosphatidylinositol 3-kinase (PI3K) pathway [35] (Figure 3B). Nef is also found to be in the extracellular compartment, leading to interference with hematopoiesis, and its excretion may be associated with exosomes [36,37]. Nef also has an anti-apoptotic effect that promotes efficient viral replication [38] and pathogenesis. Nef activates NAK (PAK2) through guanine nucleotide exchange factor Vav and the small GTPase Rac1 and Cdc42. Nef-mediated activation of PAK involves PI3K, which acts upstream of PAK, and the Nef-associated PI3-PAK complex phosphorylates the pro-apoptotic Bad protein, thus blocking apoptosis [39,40] (Figure 3C). 

#### 2.2.2. Accessory Vpr

Viral protein R (Vpr), a small basic protein (14 kDa) of 96 amino acids, is conserved in human (HIV-1 and HIV-2) and non-human (SIVs) lentiviruses. Vpr is mainly involved in the G2/M arrest of dividing cells [41,42,43], but it is also one of the virus protein members of the pre-integration complex that targets neo-synthesized double-stranded viral DNA to the nucleus, and it induces apoptosis and has some transactivation activity of HIV-1 long terminal repeats (LTR) [44]. The innate and cellular immunity of infected individuals is affected by the action of Vpr via the inhibition of IL-2 production and enhancement of glucocorticoid activity [45]. Vpr also triggers virus production by inducing TNF which activates HIV-1 expression and replication via the NFκB pathway. In the absence of Vpr, there is a delay in viral replication and disease progression as shown experimentally with SIVmac with mutated *vpr* [46,47].

Previously, several chimeric lentiviruses have been generated in our laboratory [48,49,50] by inserting the SIV *nef* and *vpr/vpx* genes separately or together in the infectious molecular genome of CAEV. All chimeras were found to be replication competent and showed increased cytopathicity in cell culture systems. The chimeric virus that has both *nef* and *vpx*/*vpr* genes was used along with the parental CAEV to inoculate newborn kids which were monitored for six months. The results showed that kids infected with this chimera have an increased persistence of viral replication in the blood mononuclear cells compared to kids infected with the wild-type CAEV. In addition, a persistent decrease in the proportion of circulating T cells was observed only in kids infected with the chimeric virus. Altogether, these data clearly provide the demonstration that the increased complexity of the CAEV viral genome is associated with the increased virulence of this virus.

### 2.3. HIV Tat Protein

**Figure 4:**
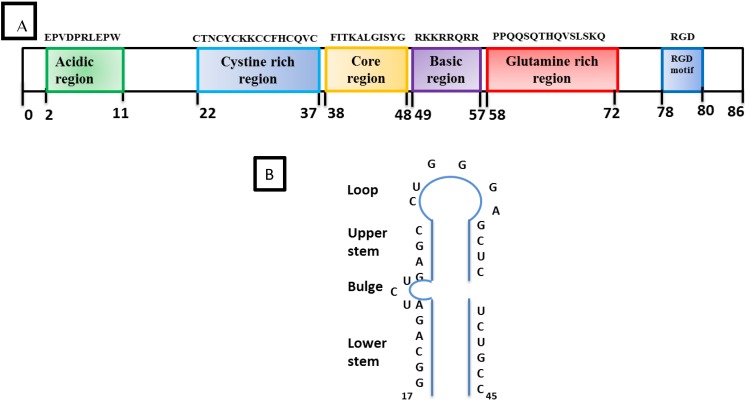
Structural organization of Tat and TAR: (**A**) The boxes in different colors indicate the position of the six different domains in the amino acid sequence of HIV-1 Tat. The Basic region from 49–57 is required for RNA binding. (**B**) Schematic representation of the trans-activation response (TAR) stem-loop structure with its different regions and the bulge structure which is essential for the high-affinity binding of Tat. The minimal sequence element required for Tat-responsiveness is from residues 19–42.

HIV-1 Tat is a 9–14 KD protein containing 86 amino acids encoded by two exons. It is one of the HIV-1 highly conserved proteins that is produced early in the HIV-1 infection. The first exon encodes the first 72 amino acids. Tat is subdivided into six functional domains (Figure 4A) including a proline and cysteine-rich N-terminal domain, a hydrophobic core, a basic region followed by a glutamine-rich region, and a C-terminal domain that contains a tripeptide RGD (Arg, Gly, Asp) [51,52,53,54,55]. In addition, there is a basic region called the protein transduction domain (PTD) which facilitates the trafficking of Tat through the plasma cell membrane. Thus, Tat can be found in the nuclear and cytosolic compartment of the cell as well as in the extracellular compartment. The primary function of Tat is to transactivate the promoter in the HIV-1 5’LTR for increased transcription of the proviral genome into full-length viral RNA [56]. 

### 2.4. Absence of SRLV Tat

Earlier studies suggested that SRLV genomes have *tat*-coding sequences upstream of the *env* gene. Furthermore, the encoded protein was found to be associated with high transactivation activity of the LTR promoter of SRLVs [57,58,59]. These data were not confirmed in the later studies and, in contrast, recent findings from our laboratory using more sophisticated and novel tools have proven that these proteins were associated with weak, if any, transactivation activities on their respective or heterologous LTRs [60]. In addition, the nucleotide sequence of the open reading frame called *tat* does not produce a protein structurally or functionally comparable to primate lentivirus Tat [61]. Historically, this open reading frame located upstream of the *env* coding sequences in the genome of SRLVs has been named *tat* because of its position rather than its structure or function; as such, it appears a misnomer. Recent studies from our laboratory clearly demonstrated that this protein was associated with activities ascribed to HIV-1 Vpr and, therefore, this SRLV open reading frame was renamed Vpr-like [60,61,62,63].

## 3. Natural History of HIV-1 and SRLV

### 3.1. Discovery of HIV-1

In 1981, AIDS was first recognized as a disease when homosexual men were reported with unusual opportunistic infections and malignancies. The principle causative agent of AIDS was a retrovirus now termed HIV-1, which was first discovered by Barré-Sinoussi *et al.* in the laboratory of Montagnier at the Pasteur Institute in Paris [4].

### 3.2. Cross-Species Infection from Monkeys to Human

HIV-1 is closely related to SIVs which have, on many occasions, jumped the species barrier from non-human primates to humans and spread successfully in the human population [64,65,66,67]. There are four phylogenetic lineage groups of HIV-1 (M, N, O, P) that were all thought to result from independent cross-species transmissions. It is now clearly established that cross-species transmission of the M and N groups of HIV-1 occurred from SIVcpz from naturally infected chimpanzees (*Pan troglodytes*) in South Cameroon, while a recent report indicated that O group originated following cross-species transmission from western lowland gorillas [68].

### 3.3. HIV Transmission in Humans

HIV-1 is generally transmitted through: (1) exchange of bodily fluids such as blood, semen, rectal fluids, vaginal fluids, breast colostrum, and milk from an infected person to recipient persons; (2) vertical transmission from mother to child either *in utero*, during delivery, or during post-natal breast feeding of colostrum or milk containing infected cells; (3) contaminated needle exchange during drug use or accidental iatrogenic exposure (reviewed in detail in [69]).

### 3.4. HIV Disease Progression 

The rate of disease progression upon HIV-1 infection is variable and depends on host and viral factor interactions. The regular pattern of progression of HIV-1 infection has been found to be punctuated *in vivo* in three phases: 1) an acute primary infection associated with seroconversion and transient illness; 2) an asymptomatic phase during which the controlled virus continues to replicate, leading to a chronic immune activation; 3) a symptomatic phase during which the virus replicates to a higher titer. At this stage, the immune system is impaired and allows the proliferation of opportunistic infections [70]. 

### 3.5. First Descriptions of SRLV Infection

A disease that is now known to be associated with lentivirus infection in sheep was first described in South Africa by Mitchell in 1915 and termed as progressive pneumonia of sheep [71,72]. The next description was reported in sheep from Montana in 1923 with severe chronic interstitial pneumonia that resulted in wasting and, eventually, death of the affected animals [73]. In Iceland, about a decade later, “maedi”, a chronic form of pneumonia, emerged in a local breed of Icelandic sheep, progressing to a fatal form associated with important weight loss and shortness of breath [74]. There was also a neurological form of disease, “visna”, associated with paralysis and wasting that affected the Icelandic sheep during the next decade [72]. Epidemiological studies showed that the appearance of Maedi/Visna diseases began in a local breed of sheep after the import of asymptomatic Karakul rams from Germany in 1933. These lambs were imported for introducing genetic diversity because of the valuable Persian lamb skins of Karakul sheep for the Icelandic industry. An eradication program that lasted nearly a decade and in which all the sheep (over 100,000) in the affected areas were slaughtered was the only way to stop the fatal loss of the local breed [75]. During the following years, intensive studies were conducted for better understanding the Maedi and Visna diseases. Virological and pathological studies under Dr. Bjorn Sigurdsson helped to conclude that a lentivirus was the etiologic agent responsible for both Maedi and Visna [6,7]. This was described as a “slow/low” virus that induces disease in infected sheep only years post-infection. Since then, the virus causing this type of infection in sheep was widely called “Maedi Visna Virus” (MVV) or ovine progressive pneumonia (OPPV) or ovine lentivirus (OvLV). 

The pathological changes that are known to be attributed to the goat lentivirus infection CAEV were reported in the late 1950s in adult goats in Switzerland [76] and about a decade later in Germany [77]. Later, Linda Cork in the early 1970s described in detail the anatomic-pathological changes associated with this disease [78]. The first isolation of the etiologic agent was simultaneously reported from the joints of an arthritic goat [79] and from the CNS of an encephalitic kid [80]. Later, the virus was isolated from goats all over the world. All lentivirus isolates from sheep and goats are referred to now as Small Ruminant Lentiviruses (SRLVs). 

### 3.6. Cellular Tropism in Vivo and in Vitro

The main cell types that support SRLV replication *in vivo* are those of the monocyte/macrophage cell lineage [8]. Unlike primate lentiviruses, SRLVs do not replicate productively in CD4+ T lymphocytes [9]. The mechanisms underlying this restriction are still not well defined. *In vivo* in circulating monocytes, the viral DNA remains silent, not producing viral proteins and, thus, latently infected monocytes largely escape immune surveillance, providing for a Trojan horse mechanism [81]. Virus replication becomes productive only upon their differentiation into macrophages following their homing in tissues [82,83,84] in different organs [85]. SRLVs can infect cells other than monocytes/macrophages, including microglia, astrocytes, and oligodendrocytes [86,87]. In cell culture, SRLVs productively infect the goat synovial membrane cells, sheep choroid plexus cells [88,89], fibroblasts [90], and epithelial and endothelial cells [91]. Unlike primate lentiviruses, SRLVs do not use CD4, CCR5, and CXCR4 molecules as receptors/coreceptors for target cell infection. However, the receptor/coreceptor used by SRLVs are still not well identified. 

HIV-1 infects predominantly the CD4+ T cells following attachment to the principal CD4 receptor and either the CCR5 or CXCR4 co-receptor. Following the entry of HIV-1 in the human body, there is a bottle neck selection of a founder virus that predominantly uses the CCR5 as a co-receptor [92,93]. This is primarily expressed on the surface of memory CD4+ T cells. The infection of such cells may lead either to productive HIV-1 replication and cell death via cytopathic effects or non-productive HIV-1 latently infected cells, depending on the activation dynamic of the host cells [94]. In addition to CD4+ T lymphocytes, HIV-1 has the ability to infect myeloid cell lineages such as dendritic cells and monocytes/macrophages [95] both *in vitro* in cultured cells and *in vivo* in infected humans [96]. In blood, HIV-1 proviral DNA is detectable in less than 1% of the circulating monocytes [97,98,99,100]. However, monocytes disseminate to all tissues of the organism where they rapidly differentiate into specialized tissue-specific macrophages [101] and persist as infected cells for a long period of time. In addition to monocytes/macrophages, epithelial and endothelial cells and fibroblasts are susceptible to *in vitro* infection with varying levels of viral replication [10,91,102,103]. The other cell types susceptible to HIV-1 infection are dendritic cells (myeloid, Langerhans, and plasmacytoid) that are antigen-presenting cells which can be targets of HIV-1 infection [104,105,106]. Dendritic cells were found to capture the virus through lectin-type receptors such as DC-specific ICAM-3-grabbing non-integrin (DC-SIGN) or sialic acid-binding Ig-like lectin 1 (SIGLEC-1), and act primarily as intermediates for virus transmission to T cells since HIV-1 replication in these lineages is limited [107,108,109].

### 3.7. Cross-Species Infection

Earlier experimental infections of sheep with the goat lentivirus (CAEV) and goats with ovine lentivirus (MVV) have provided the demonstration of the lack of species barrier for these viruses in sheep and goats [110]. Later, viruses genetically closer to CAEV were found in naturally infected sheep [111,112] and others genetically closer to MVV were found in naturally infected goats [113]. The experimental infection of mouflons [114] and calves [115] clearly demonstrated that the virus is capable of causing infection in a large variety of ruminants. The natural cross-species transmission of SRLVs in domestic and wild ruminants has been recently documented [116]; there is also evidence of mixed infections in field conditions with two closely related SRLVs. In addition, there have been descriptions of goats persistently infected with both CAEV and MVV and *vice versa* [117].

### 3.8. Natural SRLV Transmission

In adult sheep the virus is mainly transmitted by the respiratory route through the exchange of droplets when animals are housed in high density in closed facilities during the winter [118]. In newborn lambs and kids, the virus is transmitted mainly by consumption of virus-infected cells in the colostrum and milk from infected dams and female goats [119,120,121]. In early stages of small ruminant development, the gastrointestinal tract is porous, allowing infected cells to cross the epithelium barrier and invade lymph nodes and blood where they then cause infection of the peripheral blood mononuclear cells [122].

### 3.9. SRLV Viral Dynamics in Vivo

Unlike primate lentivirus infection, there are no defined phases of infection with SRLV [118,119,123]. Most infected animals remain lifelong seropositive following seroconversion and the virus is thought to cause lifelong persistence in the host monocytes where it can stay in a latent form. Following the differentiation of monocytes into macrophages in tissues, latent proviruses become transcriptionally active and productive of new infectious virus. The time between infection and seroconversion is greatly variable and can take more than two years [124]. Only a fraction of persistently seropositive animals remain productively infected and capable of transmitting the virus without showing any clinical symptoms of disease [79,118,125].

### 3.10. Acute Primary Infection, Disease Progression, and Characterization

The initial phase of HIV-1 infection in humans includes high activity of viral replication, virus genome mutagenesis, and cell death that lasts for two to eight weeks. During this stage, viral replication can produce up to 10 billion virions/day and the concentration of HIV-1 RNA in the plasma can exceed 10^7^ copies/mL [126,127]. As mentioned earlier in section 3.6, the initial phase of infection of both SRLVs and HIV-1 targets monocyte/macrophage cell lineages but, unlike SRLVs, HIV-1 also infects the CCR5+ CD4+ T cells where it mainly replicates in the gut-associated lymphoid tissues (GALT) early post-infection [128]. This productive replication is associated with the spread of the circulating virus throughout the body to establish the main reservoirs/sanctuaries of viruses in the lymphoid tissues, the central nervous system, and the genital tract [129]. The clinical symptoms during this stage can include fever, pharyngitis, lymphadenopathy, cough, rashes, myalgia, diarrhea, headache, nausea [130,131,132]. 

Following two to six months post-infection with HIV-1 in humans, a balance is reached between the rate of viral replication and induced antiviral immune defenses, leading to a steady level of viremia evaluated by HIV-1 RNA in the plasma [133,134,135]. The initial drop of CD4+ T lymphocytes stabilizes around 350–800 cells/µL, and this phase can be maintained or show progressive decline for years. This was initially considered as the asymptomatic phase. The progression towards symptomatic stages and AIDS varies greatly between infected individuals and is linked to host genetic factors and the reactivation of latent infections of other pathogens or new co-infections, but there are other potential environmental factors [136,137,138,139]. Disease progression in the absence of therapy can evolve in three distinct patterns; the normal progressors with a progressive loss of CD4+ T cells over six to eight years before developing AIDS, the rapid progressors whose loss of CD4+ T cells occurs within less than two years, and the long-term non-progressors in whom the CD4 count remains stable and the viral load is less than 50 copies/mL [140,141]. Although there is a tiny proportion of individuals, the elite controllers who, in absence of antiviral treatment, progress very slowly or do not progress, the vast majority of infected individuals (>95%) develop progressively typical HIV-1-associated pathogenesis [142,143]. The CD4^+^ T cell count progressively declines during approximately the eight years post-infection. Clinical manifestations are those associated with CDC category B symptomatic conditions such as Herpes zoster, oral Hairy leukoplakia, Listeriosis, Idiopathic thrombocytopenic purpura, Peripheral neuropathy, oropharyngeal Candidiasis, Bacillary angiomatosis, Kaposi sarcoma, HIV-associated idiopathic thrombocytopenic purpura, cervical intraepithelial neoplasia II-III, and lymphoid interstitial pneumonitis. With further disease progression and the decline of the CD4+ cell count below 200 cells/µL, the immune system further weakens and the individual shows susceptibility for CDC category C disease conditions such as Pneumocystis jiroveci pneumonia (pcp), mucocutaneous Herpes simplex, cryptosporidial/microsporidia diarrhea, esophageal candidiasis, extrapulmonary/military tuberculosis, HIV-associated wasting, and peripheral neuropathy [144,145,146]. The end-stage disease is generally associated with CD4 counts below 100 cells/µL and with systemic fungal diseases, symptomatic cytomegalovirus infection, and HIV encephalopathy, ending with the death of the infected patient. Such disease progression is, however, unlikely to occur in patients undergoing highly active antiretroviral therapy (HAART), which markedly controls viral replication to a barely detectable level and substantially prolongs the lifespan of infected patients. There is an increase of the CD4+ T cell count to around 800 cells/µL and there are no common opportunistic infections, but some patients develop degenerative diseases associated with persistent chronic inflammation in addition to the risk of virus rebound and the emergence of drug-resistant variants [114,147]. 

For SLRVs, after a long preclinical period lasting many years, animals may develop inflammatory symptoms characterized by arthritis and mastitis in adult goats and, in rare conditions, encephalitis in kids [80]. In some unique circumstances, CAEV-infected kids can also undergo CNS disease characterized by leukoencephalomyelitis in animals that are one to four months old [148]. This might result from the synergistic effects of coinfections of CAEV with other pathogens, inducing local inflammation leading to permeabilization of the blood barrier and infiltration of CAEV-infected monocytes in the CNS. Animals can also develop chronic progressive arthritis with lympho-plasmocytic synovitis predominantly in adults [149].

In sheep, the symptoms are mainly in the lungs as a result of interstitial pneumonia, in the udder (mastitis), and the CNS (encephalitis) [6,116]. The clinical symptoms described by Sigurdsson *et al.* in 1957 appear mainly in adults, including weight loss, shortness of breath with progressive respiratory distress, and the enlargement of lungs. The progression of the disease to Visna, on the other hand, causes massive demyelination in the central nervous system associated with neuronal death, including in the spinal cord, leading to progressive paralysis of infected animals [7,150].

As mentioned previously in section 3.6, SRLVs do not replicate productively in T lymphocytes and both in early and late phases of infection there is no measurable depletion of CD4+ T lymphocytes in the periphery; therefore, infected animals do not undergo immunodeficiency. Moreover, unlike the cross-species infection of SIVs that has originated pathogenic persistent HIV-1 in humans, the experimental cross-species infection of adult mouflons and newborn calves with CAEV resulted in the productive infection of animals but did not result in the increased pathogenicity of virus. In mouflons, CAEV caused productive persistent infection [114] while, in contrast, in calves, it resulted in the diffusion of CAEV in target organs and transient replication, though, after four months, there was a total spontaneous clearance of all evidence of CAEV infection [115]. This provided the first demonstration that productive lentivirus experimental infection can be naturally cleared by the host’s defenses. This last finding is promising for the development of new potential vaccine strategies to fight against lentivirus infections.

## 4. Clinical Pathogenesis

HIV-1 mucosal transmission may occur through several sites including rectal, vaginal, and/or penile and oral mucosa. Rectal transmission is considered to be the easiest mucosal route for viral acquisition [151], where HIV-1 and HIV-infected cells transgress the epithelial layer via small breaks to cause systemic diffusion of the virus following the infection of numerous target CD4+/CCR5+ T lymphocytes. This transmission is thought to be established by one founder virus tropic for CD4+/CCR5+ T cells. This founder has enhanced interaction with dendritic cells and is resistant to the IFN-γ host response [152]. Another mechanism highlighted for this route are M cells which produce mucus and form intraepithelial pockets that have CD4+ memory T cells and dendritic cells and promote the trans-epithelial transport of HIV-1 [153,154]. 

For vaginal transmission, extensive work is still ongoing to highlight the major target cells throughout the female reproductive tract (FRT). FRT is a strong barrier that effectively prevents the virus from transmission to germinal cells, so for virus entry it is likely that there is more than one site. The vagina and the ectocervix comprise multilayered epithelium, while the endocervix has only a single columnar epithelium layer [155]. In a recent rhesus macaque vaginal transmission model study, using a single round non-replicating SIV-based vector with dual marker genes, it was shown that the entire FRT including the vagina, ecto-, and endocervix, along with the ovaries and local draining lymph nodes, can contain vector transduced cells only 48 hours after inoculation. The results from this study clearly suggest that virions quickly disseminate after the multisite entry of cells throughout the FRT with a preference for infection in squamous vaginal and ectocervical mucous. This study for the first time showed that even if the infection occurs primarily in vaginal and ectocervical tissue, it can spread as far as the ovaries and local draining lymph nodes [156].

Thereafter, the brunt of the viral replication has been found to be in immune cells of the gastrointestinal tract [157,158], named the gut-associated lymphoid tissue (GALT). GALT represents the most important lymphoid organ harboring 40–80% of all T cells in the body [159,160]. Importantly, this site predominantly contains memory CD4+/CCR5+ T cells, expressing the major receptor and co-receptor for HIV-1 and SIV entry [161,162]. Infection in the GALT is extensive, reaching 60% of the mucosal memory CD4+ T cells within days post-infection and the infected cells are rapidly eliminated [163,164]. 

Intravenous transmission of the virus is faster as there is no selective barrier between virus and target cells, and the virus rapidly disseminates to all lymphoid tissues including thymus, spleen, peripheral lymphoid organs, and mucosal lymphoid tissues, with a peak of viremia at 10–14 days post infection. The productive infection is observed within two weeks in the paracortex of the lymphoid sheath in the spleen, and in the thymus medulla. Like in the mucosal transmission, the main targets of the infection are memory CD4+ T cells expressing the CCR5 receptor (reviewed in [165]).

As alluded above, progressive HIV-1 infection is associated with the progressive decrease of CD4+ T cells both in number and in function, but also, as recently shown, with continuous chronic inflammation, is initiated by a burst of pro-inflammatory cytokines (reviewed in [166]). This is followed by the chronic presence of several pro-inflammatory factors, such as IL-6, TNF, and others, leading to early immune senescence and remodeling of lymphoid tissue [167]. 

In the CNS, the late stages of HIV-1 infection can be complicated by AIDS dementia complex or HIV-associated Dementia (HAD), which is a neurological syndrome characterized by abnormalities in cognition, motor performance, and behavior [168]. Invasion of the CNS occurs both by cell-free and cell-associated virus very early post-infection [169,170,171]. This occurs following virus and virus-infected cells crossing the blood-brain barrier using complex and multiple strategies [172]. Viral replication in the CNS relies predominantly on macrophages and microglia, often leading to multinucleated cells that are due to virus-induced cell fusion. The dementia is partially if not totally due to the inflammatory effect of the virus in the brain [173]. There is absence of productive infection in neurons and oligodendrocytes, while astrocytes may under select circumstances appear infected with limited contribution to viral replication. In the era of HAART, AIDS dementia complex has been largely eliminated but is replaced by more subtle motor and cognitive impairments regrouped under the term HIV-1 associated neurocognitive disorder (HAND), which is hypothesized to be caused by the low-grade chronic inflammation and neurotoxicity of select areas of the brain. The issue is often complicated by the poor penetration of many anti-retroviral drugs across the blood-brain barrier [174].

In SRLVs, the multiple routes of infection might influence the outcome of pathogenesis. Although the main route of SRLV transmission is thought to be through ingesting contaminated milk and colostrum containing infected cells, studies have shown that various cells in the female as well as male genital tracts are susceptible to permissive infection [175]. The main clinical disease is characterized by inflammatory lesions including infiltrates of lymphocytes, plasma cells, and macrophages into various regions of the central nervous system, joints, lungs, and the mammary glands [176,177,178]. The inflammation is fueled by infected cells expressing viral antigens promoting the local activation of immune cells. This sustained activation/inflammation causes tissue damage by cytokine-mediated amplification of antiviral immune responses leading to the damage of uninfected cells (bystander effect) in the affected tissue. In the CNS, the lesions are limited to the white matter and appear as focal discolored areas, which are prominent in the spinal cord [78,148,178]. Microscopic changes are observed in the white matter with perivascular cuffing and the accumulation of mononuclear cells. In all infected kids undergoing CNS disease, the spinal cord, especially the segments in the cervical and lumbosacral parts, is involved. The CNS inflammatory disease also comprises meningoencephalitis, microgliosis, and astrocytosis in different parts of the CNS, including the spinal cord [78,179]. Lesions are generally not seen in spinal ganglia, nerve roots, and peripheral nerves. In the joints, microscopic lesions include proliferative synovitis of the joints, tendon sheaths, and bursa. As the disease progresses, fibrosis, necrosis, and mineralization of synovial membranes and the enlargement of carpi become more evident [149]. The goats gradually exhibit a poor hair coat and lose weight to become cachectic. The clinical signs include lameness and atrophy of the muscles of the affected limb. In the lungs, pulmonary lesions may vary, depending on the severity of the infection, ranging from mild congestion and interstitial pneumonia to patchy interstitial pneumonia in affected goats [78]. The clinical signs include rapid shallow respiration and increased weight of lungs; tracheobronchial and mediastinal lymph nodes are generally enlarged. In sheep, lung lesions can progress to very severe interstitial pneumonia with massive infiltrates of mononuclear cells in the interstitium causing obstruction of alveoli, which may be associated with smooth muscle hyperplasia and fibrosis of the lungs [180]. This results in the progressive reduction of the respiratory volume and may cause the death of animals [181]. In the mammary glands of infected does and ewes, extensive intralobular infiltration of mostly lymphocytes and marked lymphoid hyperplasia adjacent to lactiferous ducts have been noted [123,182]. 

## 5. Receptor/Co-Receptor Usage

As mentioned previously in section 3.6, HIV-1 interacts with the cell surface CD4 receptor and a chemokine co-receptor molecule, which triggers the structural changes in gp120, resulting in movement of the variable loop and exposure of the co-receptor binding site. When the co-receptor binds, further structural changes take place mainly in gp41 which lead to fusion of the viral and cellular membranes and internalization of the virus capsid into the cell cytoplasm. There have been seven transmembrane receptors identified *in vitro* as co-receptors for HIV/SIV that support the infection of CD4+ cells [183]. However, *in vivo*, CCR5 and CXCR4 are the major co-receptors supporting the infection [184], and HIV-1 uses either one or both co-receptors for entry.

Receptor usage by SRLVs has been studied but, to date, no receptor/co-receptor mediating entry has been clearly and reproducibly identified. Studies have suggested the mannose receptor (MR) as a potential SRLV receptor either alone or in concert with another receptor. However, cells lacking MR can be infected, suggesting that at least other receptors may be used by the virus [185,186]. *In vivo*, SRLVs have tropism for and replicate essentially in cells of the monocyte/macrophage lineage and dendritic cells in various tissues [90,187].

Compartmentalization of viral replication and evolution has been observed in both HIV-1 [188,189,190] and the SRLVs [117,191], likely due to site-specific differences in immune selection. Conserved sequences in the hypervariable V3 region of HIV-1 Env have been identified and suggested to be determinant for virus entry of macrophage tropic strains; this region also determines the replication efficiency and cell tropism [192]. In SRLVs, the Env hypervariable region V4 is structurally and functionally similar to the V3 region of HIV-1 [193,194]. The variation in the Env V4 regions gives rise to the subpopulation of viruses that colonize different organs [193]. The diverse compartments of CNS and genital tracts contain unique HIV-1 variants that are clearly distinct from the ones found in blood and lymphatic tissues [195].

## 6. HAART in HIV-Infected Patients

HAART consists of combinations of drugs like nucleoside/nucleotide reverse transcriptase inhibitors, non-nucleoside reverse transcriptase inhibitors, fusion and entry inhibitors, protease inhibitors, and integrase inhibitors. According to the last UNAIDS 2014 report, there are 35 million HIV-1-infected individuals worldwide and nearly half of them (13.6 million) now have access to antiretroviral therapy. These treatments have significantly increased the life span of HIV-1-infected individuals and virtually eliminated numerous terminal comorbidities such as Kaposi sarcoma and other opportunistic infections. While an important breakthrough in the treatment of HIV-1, these drugs fail to fully eradicate the virus infection from the body of infected, treated individuals since numbers of latently infected CD4+ T cells escape the treatment effects that are highly efficient only on cells productively replicating the provirus [196]. The discontinuation or interruption of HAART invariably results in the rebound of viremia from the latent reservoirs of HIV-1 [197,198]. Reservoirs of HIV-1 (estimated at 10^5^–10^6^ cells) are established within the first few days of infection [199]. The reservoir sites include immune-privileged organs [200] with limited antiviral immune responses (CNS and testes) or organs separated from blood via anatomical barriers that limit the access of antiretroviral therapy [201,202].

## 7. Latency and Persistence

Lentiviruses have developed multiple complex strategies to persist in the infected hosts, inducing progressive chronic diseases. Among these strategies, there is the post-integration latency of the provirus involving various heterogeneous cellular and viral mechanisms regulating the balance transcriptional active/latent proviruses that are still not fully understood [203,204,205,206]. HIV-1-associated latency has been the most studied, particularly in the context of the HIV-1 cure after HAART treatment [207,208]. After virus entry and reverse transcription, HIV-1 double-stranded DNA can either stay episomal or become irreversibly integrated preferentially in transcriptionally active sites of the host’s cell chromosomal DNA. However, a limited number of proviruses also integrate in transcriptionally inactive sites and remain transcriptionally silent in a state called viral latency. There are two main types of latency. The first is pre-integration latency in which viral DNA remains unintegrated located in the cytoplasm for a few days in the form of preintegration complex (PIC) and will eventually be degraded over time due to the cells' low metabolic rate. However, if the cell is activated before the decay of the PIC, it will then integrate and undergoes productive infection. The second type is post-integration latency whereby proviral DNA integrated into the host genome remains transcriptionally silent. Several factors and mechanisms have been associated with post-integration latency. The most prevalent mechanisms of latency currently recognized are [203,209]:

- Absence of Tat, or non-functional transactivation activity of Tat [210].
- Epigenetic regulations/chromatin remodeling by post-transcriptional modifications (hypoacetylation or trimethylation) [211].
- Insufficient levels or lack of nuclear host transcription factors (SP1, NF-kB) [212,213].
- Expression of transcription factor complexes with negative regulatory activities (YY1, CBF-1/RBP, APOBEC3G) [214,215].
- Influence of integration sites and provirus orientation on HIV-1 transcription efficiency [216].
- Unproductive control of viral RNA splicing, due to absence of Rev and innate host antiviral processes [217].

The viral regulatory protein Tat is thought to be one of the major factors in the regulation of HIV-1 latency. The absence of Tat results in a repressive chromatin environment that blocks the elongation of transcription [218,219].

In SRLVs, the viral DNA can remain in transcriptionally silent until the circulating blood monocytes mature into macrophages after their localization in tissues. This mechanism is independent of Tat, since SRLV genomes lack the gene encoding this protein [60]. In HIV-1, the proviruses in monocytes could be associated with a minimal amount of viral proteins without active expression of the proviral genome, thereby escaping detection and killing by the immune system; however, like SRLV-infected monocytes, upon entry in various tissues their rapid maturation into tissue macrophages is associated with increased expression and the release of fully infectious viral particles [8,220]. In SRLVs, viral persistence is fueled mainly by the lack of or low-grade viral replication in infected circulating monocytes that disseminate productive replication of the virus in various tissues. However, SRLV replication in macrophages can be also restricted by a number of posttranscriptional blocks [103,221,222]. 

## 8. Molecular Biology of HIV-1 Latency

### 8.1. Molecular Mechanisms of the Transcription Involving Tat in HIV/SIV 

**Figure 5:**
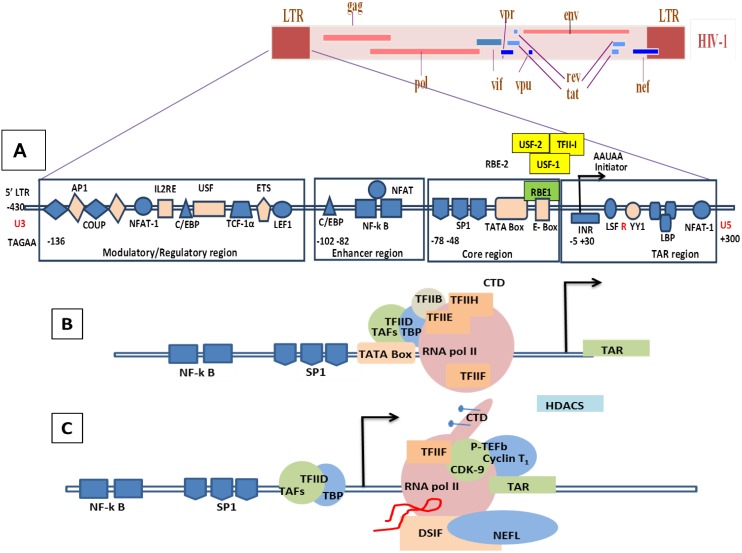

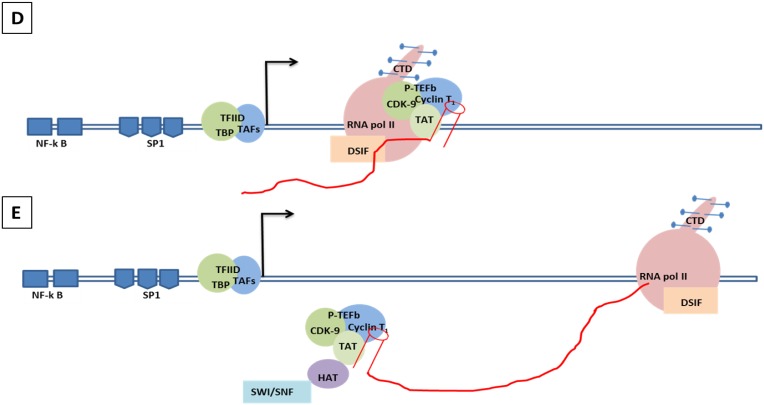
Genomic organization of HIV-1 and mechanisms of LTR transactivation. (**A)** The modulatory region consists of transcription factors such as COUP, AP1, NFAT-1, IL2RE, C/EBP, USF, TCF-1α, ETS and LEF1. The enhancer region has the NF-κB and C/EBP, and the core region consists of three tandem SP1 sites and the TATA box and E-box to which RBE1 and RBE2 (USF1, USF2, TFII-I) binds. Downstream to the TATA box is the initiator element from the TAR region. A weak transcription starts at -430 at U3 with TAGAA and is enhanced at the downstream TAR region at the +1 AAUAA polyadenylation site. (Not all the transcription binding sites are shown here). (**B**) Initiation stage: the cellular transcription factors TAFs and TBP (TAFs + TBP + TFIID) are recruited which, along with the other components of the basal transcription, help in recruiting the RNA polymerase holoenzymes. The CDK7 kinase presents the CTD of the RNA polymerase in the TFIIH phosphorylates. (**C**) The hypo-phosphorylated state of the CTD correlates with the low processivity of the RNA polymerase enzyme complex. Due to hypo-phosphorylation, and in absence of Tat, there is mainly production of short RNA. Furthermore, DSIF and NELF that bind to the RNA pol II inhibit the transcriptional elongation. (**D**) CDK9 phosphorylates the CTD and, along with cyclin T1, they produce the complex P-TEFb (positive transcription elongation factor b). TAT is recruited and binds to the bulge sequence of the TAR RNA. The hyper-phosphorylation of CTD is induced by the binding of the P-TEFb to the TAR, resulting in dissociation of DSIF and NELF. HATS and SWI/SNF, which induces acetylation of the nucleosomes, are recruited and remove any blocks due to epigenetic modification and support elongation. The acetylation of Tat creates a binding site for P300/CREB binding protein-associated factors (PCAF) which enhances the transcription elongation.

To date, there is no identified viral negative factor that represses HIV-1 transcription to undergo latency. Factors that were thought to be negative regulators in initial studies were not confirmed in more recent studies. The transcription of HIV-1 is tightly controlled by powerful feedback mechanisms of viral and cellular factors that act like molecular switches to regulate the expression of HIV-1 RNA transcription. HIV-1 transcription comprises two phases: first, an initiation step, and then an elongation step. The core promoter region in the U3 region of the LTRs includes several important motifs for the binding of factors required for the transcription, including a TATA box element, three SP-1 sites, and an initiator element (Figure 5A) [223,224,225,226]. Each of these core promoter elements contributes to the binding of the initiation complex TFIID. During the initiation phase, several factors such as the TATA box binding protein (TBP) and TBP-associated factors (TAFs), which interact with the TATA box and SP-1, are recruited by the promoter region to initiate the transcription complex [227]. A recent study shows the presence on an E-box motif (RBE1) within the core promoter that is implicated in transcriptional activation. RBE1 is a binding site for the RBF-2 transcription factor complex (USF1, USF2, and TFII-I), previously shown to bind an upstream viral element, RBE3. The results indicate that RBE1 is a *bona fide* RBF-2 binding site and that the RBE1 and RBE3 elements are necessary for mediating proper transcription from the HIV-1 LTR [228]. In another new study, it has been shown that CTGC motifs flanking the HIV-1 TATA box are required for Tat transactivation in living cells and the correct formation of pre-initiation complexes of HIV-1 (PICH); this complex contains the general transcription factor TFIIA that binds the HIV-1 core promoter formation *in vitro*. The binding of known core promoter binding proteins AP-4 and USF-1 was found to be dispensable for Tat function. The transcriptional response element (TAR) RNA prevented the stable binding of PICH-2. The impact of TAR on PICH-2 specifically required its bulge sequence, which is also known to interact with Tat [229]. A study by Jensen *et al.* using an *in vitro* model of a HIV Tat-mediated positive-feedback loop demonstrated that fluctuations in viral Tat-transactivating protein levels generate integration site-dependent, stochastically-driven phenotypes, in which infected cells randomly ‘switch’ between high and low expressing states in a manner that may be related to viral latency. Furthermore, in an extended model, they designed a forward genetic screen that systematically identifies the genetic elements in the HIV LTR promoter that modulate the fraction of genomic integration sites that specify ‘switching’ phenotypes. In this experiment, it has been shown that specific mutations in the core promoter regions, including Sp1 and TATA transcription factor binding sites, can increase the switching fraction several fold. Using the single-cell system experiments with computational modeling, they could further investigate the mechanism of switching-fraction enhancement for a selected Sp1 mutation. Altogether, these experiments demonstrated that mutations in the Sp1 site impaired not only Tat-induced transactivation of expression, but also the basal expression in the absence of Tat. Computational analysis demonstrated that the observed change in basal expression could contribute significantly to the observed increase in viral integrations that specify a switching phenotype, provided that the selected mutations affected Tat-mediated noise amplification differentially across genomic contexts [230].

Upstream of the core promoter region there is the regulatory region of HIV-1 LTR, which has binding sites for the cellular transcription factors nuclear factor of activated T-cells (NFAT), AP-1, p65/p50 nuclear factor kappa-light-chain-enhancer of activated B cells (NF-kB) and Signal Transducer and Activator of Transcription 5 (STAT5), which orchestrate HIV-1 expression. The enhancer region of the regulatory promoter region consists of the NF-κB binding site, which has been studied extensively. Any mutation in one or more core promoter sites or regulatory promoter elements or enhancer regions leads to strong impairment of both basal and Tat-dependent transcription [231,232,233,234,235]. The key factor in the initial stage of the transcription of the HIV-1 LTR promoter by RNA polymerase is the phosphorylation of the carboxyl terminal domain (CTD) [236]. The CTD phosphorylation renders the RNA polymerase highly processive. The phosphorylated polymerase, once cleared from the promoter region, is then able to transcribe through the TAR region [237] (Figure 5B).

The initial phase in the elongation of HIV-1 transcription produces a low-level of viral gene expression which is a result of the hypo-phosphorylated condition of RNA pol II and activity of the negative elongation factor, 5,6-dichloro-1-beta-D-ribofuranosyl-benzimidazole sensitivity-inducing factor (DSIF), and negative elongation factor complex (NELF) [238,239,240]. This stage allows the initial production of small amounts of Tat, which plays an important role during the RNA Polymerase II elongation phase. Recent studies suggest that DSIF binds the elongation complex via association with the nascent transcript and subsequently recruits NELF [241,242]. In the case of the HIV-1 provirus, the recruitment of NELF may be further enhanced by the ability of NELF-E to bind directly to the TAR region of viral RNA [243] (Figure 5C). Unlike other transactivation factors, Tat does not bind to the DNA, but it interacts with the TAR element of the genomic viral RNA. TAR is a stem loop structure containing three nucleotide bulges (23–25 residues) and a loop of six nucleotides (30–35 residues) (Figure 4B). The bulge structure in TAR is the high affinity site for Tat binding [244]. During the elongation phase, which is a Tat dependent phase, Tat binds to TAR via its arginine-rich motif (ARM) and facilitates binding of the positive transcription elongation factor b (P-TEFb) [245,246], which is comprised of cyclin-dependent kinase 9 (Cdk9) and cyclin T1 [247,248,249]. The Bromo-domain protein 4 (Brd4) stimulates the kinase activity of P-TEFb for phosphorylation of the CTD of RNA polymerase II over basal levels. [250]. As a result, the HIV-1 transcription rate is enhanced several-hundred-fold, thereby producing a high level of viral gene transcripts [219,251] because P-TEFb phosphorylates subunits of the NELF and DSIF to release the RNA polymerase II activity preventing its pausing on the HIV-1 promoter [252,253] (Figure 5D,5E). For more details please refer to the following reviews on HIV-1 Tat transcription [206,209,254,255,256,257,258,259,260,261,262,263].

### 8.2. Molecular Mechanism of Tat-Independent Transcription in SRLVs

**Figure 6:**
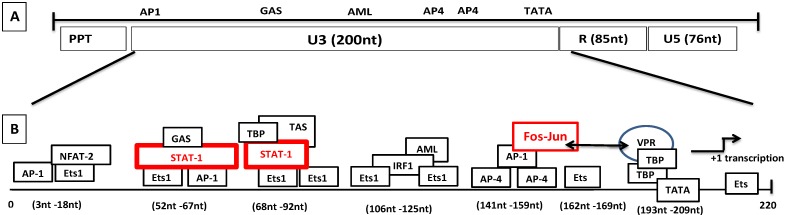
Genomic organization of SRLVs and mechanism of LTR transactivation independent of Tat. (**A**) Various regions of the SRLV LTR are distinguished. (**B)** The transcriptional elements in U3 consist of TATA box, Ets-1, Stat-1, AP4, GAS, AML, NFAT-2, IRF-1, and AP-1 sites. The proposed transactivation pathway suggests that IFN-γ activates STAT-1 phosphorylation. Phosphorylated homodimer binds to the gamma activation site (GAS) while tumor necrosis factor alpha (TNF-α) activates the transcription by binding to the 17-nucleotide TNF-activated site (TAS) within the 70-nucleotide repeats. Vpr interacts with the Jun protein of the Fos-Jun complex bound to the AP-1 site and also interacts with the TATA box-binding proteins TBP, which interact with the TATA box.

SRLV genomes lack both *tat*-coding sequences and its TAR target motif [60,61]. Earlier publications [58,264,265,266,267] reported interactions of LTR promoters with a protein called “Tat” (Vpr-Like) produced from an open reading frame whose position in SRLV genomes coincided with that of *tat*-coding sequences of primate lentiviruses. These publications reported the following findings:

(1). Vpr-like protein mediates transcription activation of SRLV genomes and this is dependent on the presence of AP-1 and AP-4 sites located in the U3 region of SRLV LTRs. The proximal AP1 site to the MVV TATA box promoter is the most important for transcription, though SRLV Vpr-like protein does not bind the AP-1 site [264,268].
(2). SRLV Vpr-like protein structure has three domains including: (a) an N-terminal acidic and hydrophobic domain, presumably the activator domain, which interacts with the TATA binding protein (TBP) *in vitro* [59,266]; (b) a central Leucine-rich domain that can interact with FOS-Jun-specific proteins in the AP-1 sites [266]; (c) a C-terminal Cysteine-rich domain which might direct protein dimerization. An interaction of the SRLV Vpr-like protein with the TBP from the TFIID complex was shown to bind to the TATA box via its activator domain, in addition to the proximal AP-1 binding to the Leucine-rich domain. These interactions allow a stabilization of protein complexes located at the viral promoter, and therefore induce an increase of viral gene transcription as illustrated in Figure 6.

Studies by Harmache *et al.* [269] showed that the *tat* gene of CAEV was fully dispensable for viral replication *in vitro* and *in vivo*. Three different infectious molecular clones of CAEV bearing three distinct mutations in the *tat* gene were used in this study to compare the replication kinetics after transfection or infection of cultured goat synovial membrane cells and infection of blood-derived mononuclear cells or macrophages. The results showed that all mutants replicated at an efficiency similar to that of the wild type. The properties of wild type and *tat* mutant viruses were also compared *in vivo* by injecting the infectious molecular clones bearing the proviral DNAs directly into the joints of goats. Animals seroconverted between 27 and 70 days post-inoculation, and the CAEV mutant in *tat* was isolated from blood-derived macrophages, confirming that *tat* was dispensable for productive replication of CAEV *in vivo* as well.

Recent data clearly demonstrated that *tat*-encoded protein is not only structurally different, but also has no transactivation activity comparable to HIV/SIV Tat [60,61]. Further studies found instead that this protein was associated with functions similar to that of HIV-1 Vpr [61,62,63]. These studies compared the trans-activation activity exerted by MVV, CAEV, and HIV-1 Tat proteins in a variety of cell lines. The sub-cellular localization of MVV and CAEV Vpr-like protein was also investigated. None of the SRLV Vpr-like proteins tested induced the transactivation of SRLV or HIV-1 LTR promoters. Unlike HIV-1 Tat, the SRLV protein was found to be incorporated into the virions. The results also demonstrated that this lack of transactivation was not associated with the inability of this protein to enter the nucleus of expressing cells. The SRLV Vpr-like protein from the MVV strain, however, induced a very modest (two- to three-fold) upregulation of SRLV LTRs. However, in similar conditions, HIV-1 Tat *trans*-activated HIV-1 LTR over 60-fold [60,61]. 

The studies suggest that the primary role of the so-called Vpr-like protein in SRLV is not the transactivation of the LTR promoter similar to that of the primate lentiviruses HIV and SIV. Further studies demonstrated that this protein is structurally and functionally related to Vpr. The mechanism of transactivation with SRLV Vpr-like protein involves an interaction with Jun protein from the Fos/Jun complex bound to the AP-1 site located in viral LTR; this mechanism is functionally very close to the HIV-1 Vpr which it interacts with in the Sp1 located in viral LTR [61,62,63].

In the pursuit of exploration of other molecular pathways which lead to the consecutive transcription of the CAEV promoters and transcription factors, various studies were performed by Murphy *et al.* in the human monocytic U937 cell line expressing the SRLV genome. These studies suggested that the tumor necrosis factor alpha (TNF-α) and granulocyte-macrophage colony stimulating factor (GM-CSF) activate the CAEV transcription. They used SP600125 to block the Jun N-terminal kinase (JNK) and the cells were subsequently treated with cytokines. SP600125 blocked the previously described transactivation pathway through AP-1 [270], yet the SRLV transactivation was observed. Therefore, they proved that the TNF-α and GM-CSF could induce the activation of CAEV promoter independent of AP-1. Furthermore, using a set of sequentially deleted mutants, they found that elements within the 70-bp repeat in the U3 region are involved in the activation by TNF-α [270]. In a similar work performed previously by Sepp *et al.*, they showed that the γ-interferon activation site (GAS) element from the 70-bp motif is adequate for the responsiveness to IFN-γ using a heterologous minimal promoter. They also showed that the binding of the nuclear factor to the GAS element is inhibited by antibodies directed against the signal transducer and activator of transcription STAT1 (p91/84) [271]. In a later work reported by Murphy *et al.*, the authors identified a sequence of 17 nucleotides called TNF-activation site (TAS) which is located within the 70-bp repeat of the U3 region. They also found other sequences within the U3 region required for the IFN-γ activation. However, additional work is needed to establish whether STAT1 binding to the U3 site is involved in the activation of SRLV promoter through TNF-α and GM-CSF regulation.

Data from experiments in these two reports show that the TAS sequence in the SRLV LTRs is essential for TNF-α and IFN-γ to activate STAT-1 phosphorylation and bind to GAS. The STAT-l signaling pathway was shown to play an important role in the regulation of viral promoter activation through IFN-γ [271,272] and GM-CSF [273]. It is also interesting to know that IFN-γ leads to the activation of CAEV LTR through the STAT-1 signaling pathway [271] in monocytes, and STAT1 also plays an important role in the differentiation of monocytes to macrophages [274]. Interestingly, natural deletions in the U3 70-bp, *vrp*-like accessory gene and *dUTPase* resulted in highly attenuated genotype E strains of SRLV isolated in Italy [275]. Furthermore, the low pathogenicity and variable virulence can be also due to the deletions and/or mutations of the replication enhancer factors of the LTR, such as AP1, AML, TNF-α, and IFN-γ responsive elements [276,277]. While in SRLV strains from sheep the viral promoter sequences in LTR were found to determine the cell tropism [278], those sequences of strains from goats were not [279,280]

In a serendipitous experiment of nature as quoted by Dr. Murphy, his group isolated SRLV (CAEV-MA) from the mammary glands and from the carpal synovium (CAEV-JT) of the same goat. Sequence analyses in viral promoters revealed that the CAEV MA had a rare loss of GAS that was replaced by 5’-ACTCGAGGGTCTAGGA-3’ [281]. Earlier studies of SRLV-infected goats showed a heterogeneity of molecular clones with different promoters in different anatomical locations [279], but it was the first time that they found the rare mutation in the same goat. Using an assay based on β-galactosidase activity measurement, they found that the mean of basal promoter activity of CAEV-MA was inferior to that of CAEV-JT (mean OD of 0.389 ± 0.083 *vs.* 007, *p* < .0001, respectively) [281].

### 8.3. Role of Epigenetics and Transcriptional Regulation Factors in Modulating HIV-1 Latency 

In general, eukaryotic DNA that is not in a transcriptionally active form is tightly packaged around nucleosomes. Each nucleosome is composed of octamers of histone proteins having pairs of H2A, H2B, H3, and H4 molecules densely packed to form the chromatin. Any change in the structure of the chromatin markedly affects gene expression. The complex of DNA/histones undergoes various post-translational modifications like acetylation, methylation, and phosphorylation, leading to modifications of the structure of chromosomes [282,283,284,285,286]. Histone modifications also contribute to the transcriptional regulation by the transcriptionally active euchromatin or transcriptionally repressive region heterochromatin which inhibits the transcription.

The post-integrational event of HIV-1 provirus into the host genome is followed by interactions with two nucleosomes, nuc-1 and nuc-0, on the LTR region. The position of nuc-1 plays an important role in controlling gene expression [287,288]. The cellular transcription factors, such as Yin Yang-1 (YY1), LSF, c-Myc, Sp-1, and AP-4, recruit histone deacetylases HDACs to the HIV-1 promoter, leading to a block of the transcription activity leading to latency [215,289,290,291,292,293,294]. The classical NF-kB complex has two subunits, p50 and p65, and this heterodimer is a potent activator of transcription. However, p50 can also generate homodimers that repress HIV-1 transcription by recruiting the HDAC to the LTR promoter. Knockdown experiments of p50 result in the reinitiation of RNA polymerase recruitment [293,295]. 

Histone acetyltransferases (HATs) such as E1A binding protein p300/CREB-binding protein (p300/CBP), P300/CBP-associated factor (PCAF), and GCN5 histone acetyltransferase may be recruited to the HIV-1 promoter. These HATs, which are critical for the acetylation of histone tails, interact with Tat, leading to recruitment of the chromatin remodeling complex SWI/SNF/BAF that opens/relaxes the chromatin and enables the transcription elongation by displacing restrictive nucleosomes [296,297,298,299]. 

There are also post-transcription modifications that are implicated in the regulation of Tat-induced latency [300]. Tat is necessary for sustained transcription of the HIV-1 LTR and suboptimal levels of Tat can lead to latency. Lack or minimal production of Tat can be due to the lack of cellular factors or due to mutations on the Tat/TAR axis. There are post-transcriptional modifications of Tat that control the interactions of Tat with P-TEFb and TAR. The dissociation of Tat from TAR and P-TEFb is due to the demethylation of Lysine 51 by Lysine-specific demethylase 1A (KDM1A/LSD1)/REST corepressor 1 (CoREST) and the acetylation of Lysine 50 mediated by Lysine acetyltransferase p300/KAT3B while, in contrast, Tat activity is enhanced by the methylation of Lysine 51 by Lysine methyltransferase SET7/9/KMT7 [301,302,303,304,305]. The transcriptional activity of pTEFb is reduced by acetylation of CDK9 of pTEFb, by the histone acetyltransferases hGCN5 and PCAF, leading to HIV-1 latency [306]. Moreover, efficient activity of Tat is dependent on the methylation of Lysine 51 by KMT7 that results in enhanced Tat binding to its target TAR in the 5’ extremity of the genomic RNA. Acetylation of Tat by PCAF on Lysine 28 is essential for recruitment of pTEF at the 5’end of viral RNA for an efficient elongation of RNA transcription. Sirtuin-1, a specific Tat deacetylase, increases the amount of unacetylated Tat in number [296].

Histone methylation, another epigenetic modification, also plays a role in regulating the latency of HIV-1. The histone methyltransferases (HMTs) such as Enhancer of zeste homolog 2 (EZH2) and Euchromatic Histone-Lysine N-methyltransferase 2 (EHMT2), also known as G9a and Histone-Lysine N-methyltransferase (SUV39H1), are involved in regulating HIV-1 transcription by inducing H3 methylation at Lysine 9 (H3K9) and Lysine 27 (H3K27). The studies by Karn’s group have demonstrated that EZH2 is a critical enzymatically active compound of the polycomb repressive complex 2 (PRC2), through which H3K27 trimethylation takes place [307]. The knock-out of EZH2 induces the loss of H3K27 trimethylation which leads to latency. SUV39H1 is an H3K9 methyltransferase that initiates the heterochromatin formation by interacting with HP1 and induces latency [204,308,309]. 

Further details on the epigenetic mechanism and post-translational modifications associated with HIV-1 latency and activation of transcription are reviewed in following [254,262,310,311,312].

### 8.4. Latency and HIV Cure

Latently infected cells are defined as the cells containing the integrated HIV-1 DNA that are transcriptionally silent but retain the capacity to produce infectious virus upon activation. These latently infected cells reside in viral reservoirs in different anatomical locations. They consist of different cell types that have stable kinetic properties and allow persistent virus replication. In the HAART era, most of the latently infected cells were abundantly found in the sanctuaries where the HAART drug penetration was limited. Theoretically, a cure can be either functional or sterilizing. A functional cure aims at permanent viral suppression following the interruption of therapy to levels that prevent immunodeficiency and transmission, while a sterilizing cure aims at the complete eradication of the viral population from the whole body [313].

Several therapeutic strategies to cure HIV-1 infection have been proposed, targeting the latently infected CD4+ T cells (reviewed in [314,315,316]).

- Reactivation of latent virus while patient is on HAART using HDAC inhibitors, vorinostats (SAHA), valproic acid, [317] “non-oncogenic” phorbol ester (bryostatin, prostratin), and cytokine IL-7 [318]. Induction of viral replication of proviruses in cells from latent reservoirs and clearance is the most promising “Kick and kill” strategy.
- Excision and removal of the proviral DNA [319,320,321],

Strategies that aim to eradicate the virus without targeting latent reservoirs:

- Creation of HIV-1 resistant cells (SB 728-T) [322,323],
- Gene therapy (CCR5Δ32/Δ32) [324,325,326] and Elite suppressors (ESs) model [327,328],
- Reversal of immune exhaustion (PD-1 antibodies) [329],

#### The Unique Case of Possible Cure

The Berlin patient was infected with HIV-1 and was on ART for more than 11 years before being diagnosed with leukemia at age 40. The curative intervention included an acute myeloid leukemia (AML) intensive conditioning regimen, including chemotherapy, antithymocyte globulin, and total body irradiation, with allogeneic hematopoietic stem cell transplantation (HSCT) from a CCR5 D32 homozygous donor. These interventions have eliminated most of the patient’s immune cells, including latently infected cells, and engrafted cells could not be infected with residual virus if any remained. This patient is established “aviremic”, and even seven years after HSCT and extensive analyses, there is no HIV-1 detected [325,326,330]. With a similar HSCT procedure, two other patients, Boston patient 1 and Boston patient 2, were intervened by reduced intensity conditioning and allogeneic HSCT from CCR5 wild-type donor. They were on ART for 4.3 years and 2.6 years, respectively, after HSCT, but they had detected a rebound after 84 days and 225 days, respectively, after ART interruption [331,332].

A case of “functional cure” has been recently reported in the International AIDS Society’s 8th conference on HIV-1 pathogenesis, treatment, and prevention. Under the French Pediatric Cohort led by Dr.Asier Saez-Cirion, an ART treatment was initiated soon after the birth of a baby girl who continued the treatment for six years and then stopped. The young woman, now 18.5 years old, has been in remission for 12 years. The early antivirals might have limited and reduced the formation of the reservoirs and protected the immune system [333]. This is the only case so far with such a long remission in children. Earlier, the “Mississippi baby” who received an early postnatal ART became aviremic for a period of 24 months. However, after cessation of HAART at 18 months, a rebound was detected, thus suggesting that the early ART treatment in the infected infants did not completely eliminate the reservoirs [334]; however, new studies have proved otherwise. In another long-term successful remission in the “VISCONTI cohort” study in adults, there are 14 post-treatment controllers (PTCs). They were initiated with the ART soon after infection and were on the treatment for at least three years before interruption. The study found that PTCs were able, after therapy interruption, to keep and in some cases further reduce a weak viral reservoir [335].

## 9. Sites of Latent Reservoirs

### 9.1. Cell Lineages

The primary cell targets of HIV-1 infections (discussed in detail in section 3.6) include the CD4+ T lymphocytes [336,337] and monocyte/macrophage lineages [338], while other cells were found to be capable of getting infected with HIV-1 including natural killer, CD8+, B and follicular dendritic cells.

#### 9.1.1. CD4+ T Lymphocytes

In normal conditions, the majority of the CD4+ T lymphocytes are not activated (naïve and resting memory cells comprising central memory T_CM_ and transitional memory T_M_) [339]. Some studies have shown that non-activated CD4+ T cells can be permissive to HIV-1, and target naïve T cells might have a low level of CCR5 expression [340,341,342]. It is estimated that only approximately 1 × 10^7^ resting latently infected CD4+ T cells are present in blood and lymph nodes [343]. During HAART treatment, latent CD4+ reservoirs escape the efficient depletion of infected activated T cells and their progressive depletion occurs only slowly, depending on cell activation and cell death. The half-life of latently infected resting CD4+ T cells is estimated to be around 44 months [344] and, thus, mathematical modeling of eradication, assuming a constant rate of reactivation/elimination of the entire reservoir of 10^6^ cells, would require 73 years [345,346]. Therefore, resting CD4+ T lymphocytes are considered the leading cell type in the HIV-1 reservoir [347]. SRLVs do not infect T lymphocytes productively or latently, therefore this cell lineage is not site of a SRLV reservoir. 

#### 9.1.2. Monocytes and Macrophages

As such, monocytes/macrophages can act like a viral reservoir, some of which are immune-privileged sanctuaries such as the CNS where they mature into microglia, perivascular and meningeal macrophages, and choroid plexus [348]. Infected microglia and perivascular macrophages induce clinical pathogenesis of HIV-1-associated dementia [349]. In general, virus production from monocytes/macrophages is markedly lower than from the CD4+ T lymphocytes [101]. Nevertheless, in addition to truly silent reservoirs, these cells may support a continuous low level virus production throughout their life since, unlike T cells, they are less sensitive to cytopathic effects induced by virus infection [101]. Gut-associated macrophages of *lamina* *propria* differentiated from blood monocytes could represent an important HIV-1 reservoir [350,351]. The role of monocytes and macrophages is also reviewed in detail in the following reviews [312,352,353,354]. SRLV in sheep and goats would be an excellent model to specifically study the mechanisms involved in the latency associated with these cells in absence of that associated with the CD4+ T lymphocyte reservoir; unfortunately, there are only limited studies. 

#### 9.1.3. Dendritic Cells

Their potential implication as players in HIV-1 latency/reservoir was demonstrated by the mature myeloid dendritic cells located in the lymph nodes which can harbor and transmit virus shielded from immune recognition for extended periods of time [355]. There have been reports that SRLVs could also infect and replicate in dendritic cells, which may also play a role in virus dissemination into lymphoid tissues, but it seems to be of low significance [90,356].

### 9.2. Anatomical Reservoirs

Among the anatomical reservoirs for HIV-1 there are two main categories that deserve special attention: (i) immune-privileged sites in which antiviral effector mechanisms are inhibited and/or (ii) sites that are inaccessible or poorly accessible to drugs used in HAART. Examples of such reservoirs are the CNS, lymphoid organs, the genital tract, and lungs. 

#### 9.2.1. CNS 

CNS is protected by the blood-brain barrier which is highly selective in the exchange between blood and the brain tissue compartments, thus creating an immunological micro-environment. The tight epithelium of the choroid plexus restricts the flow of the molecules and cells into the cerebrospinal fluid (CSF), thus creating an effective barrier which prevents the passage of most HAART drugs to the CNS. The HIV-1 reservoirs in the CNS are the microglia and, to a lesser extent, the cortical and basal ganglia-derived astrocytes in which HIV-1 DNA has been detected [357,358,359]. Microglia and perivascular macrophages produce neurotoxins and cellular factors that induce clinical pathogenesis of HIV-1-associated dementia [129,170,360,361].

In SRLVs, the endothelial cells of the vascular system of the CNS have been shown to facilitate viral entry into the CNS [10]. The virus in the CNS causes lesions and the obvious signs of meningitis along with the destruction of the myelin sheath [362]. Proviral DNA has been located in macrophages, microglial cells, astrocytes and oligodendrocytes, the ependymal epithelium, and the choroid plexus. Positive areas are also found in the spinal cord, in endothelial cells of small blood vessels of goats and kids naturally infected with SRLVs [87]. Apart from the Icelandic research, MVV is mostly associated with the pulmonary and mammary lesions, but the study done by Benavide’s group described clinical cases in 12 Assaf sheep from Spain in which the CNS lesions were only confined to the spinal cord [363]. In another study on 64 sheep with natural SRLV-associated meningoencephalitis, they found lesions particularly in the cerebellar peduncles (non-suppurative meningoencephalitis), followed by the corpus callosum, hippocampus, and thoracic spinal cord [362]. These studies are cases of CNS alterations in sheep which are not the common pathogenesis currently seen in SRLV-infected flocks.

#### 9.2.2. Lymphoid Organs

Lymphoid organs contain 98% of the lymphocytes in the body, and thus in patients treated with HAART, HIV-1 can be detected in all primary and secondary lymphoid tissues such as tonsils, bone marrow, GALT, spleen, lymph nodes, and thymus. The GALT is the tissue that contains the largest portion (40–60%) of lymphocyte populations and is also one of the largest reservoirs of macrophages [364,365]. In infected individuals, the GALT generally has half- to one-log more HIV-1 RNA molecules than in the peripheral blood mononuclear cells (PBMC) [158,366]. This increase is mainly due to the constant presence of activated CD4+ T cells leading to replication of the viruses even during HAART. Thus, while not immunologically protected or necessarily preventing HAART access, the GALT serves as a reservoir by the sheer number of activated viral targets. 

In SRLV-infected animals, provirus DNA was detected in classical peripheral lymphoid tissues (spleen, lymph nodes, thymus, bone marrow, PBMCs, and the lungs) of experimentally and chronically infected goats [102,367,368]. Although lymph nodes are considered an important viral reservoir, they do not appear to be sites of intense viral replication. Indeed, multi-spliced mRNA has not been shown to be present in samples from all goats, whereas un-spliced mRNA has been detected from lymph nodes, spleen, bone marrow, and /or PBMC [369]. 

#### 9.2.3. Genital Tract

The genital tracts of both males and females are considered to be anatomical reservoirs. The testis in particular represents an immunologically privileged sanctuary of HIV-1, which additionally also restricts the accessibility of HAART drugs [370,371,372,373]. This may result in the presence of HIV-1-infected monocytes and T cells in seminiferous tubules and the interstitium of the testis, as well as in the semen [374]. Earlier works have reported HIV-1 infection of the spermatogonia, spermatocytes, spermatids, and residual germ layer [375,376,377,378,379]. In women, the viral replication is active in the submucosa of the cervix and HIV-1 has also been detected in the epithelial and stromal cells of the uterus, fallopian tubes, and ectocervix. HIV-1 DNA was found in cervicovaginal fluids, with cervical biopsies during HAART suggesting potential limited drug penetration [380,381,382]. 

Studies in SRLVs have clearly demonstrated that goat uterine epithelial cells are susceptible to SRLV infection *in vivo* [383]. Also, SRLV proviral DNA has been identified using PCR in tissues of the genital tract (uterus, oviduct, and ovary) [175] and *in vitro*, in granulosa cells, oviduct epithelial cells [384,385], and early goat embryo cells [386]. Recent studies also demonstrate that cells of the buck genital tract are targets of SRLV infection and are thus a potential reservoir that may shed infectious SRLV into the semen of infected animals [387,388,389]. The work conducted by Fieni’s group demonstrated that the first four washes were sufficient to remove infectious CAEV and infected cells in the semen so that SRLV-free embryos can be produced by *in vitro* fertilization (IVF) using spermatozoa contaminated *in vitro* by SRLV [390].

#### 9.2.4. Lungs and Kidney

HIV-1 is positively detected in alveolar macrophages, interstitial macrophages, and lymphocytes. Alveolar macrophages harbor HIV-1 even in patients under HAART with undetectable plasma viral loads, showing that they are a potential reservoir for the virus [391,392]. Additionally, phagocytosis and immune functions are impaired due to HIV-1 replication in macrophages, which increases the risk of lung infection with other pathogens [393]. The generally low levels of replication in alveolar macrophages and the presence of alveolar lymphocytes can be rapidly augmented in lungs due to inflammatory responses to opportunistic infections. In addition, HIV-1-infected individuals are significantly more susceptible to long-term HIV-1-associated complications such as chronic obstructive pulmonary disease (COPD) [394] (reviewed in detail in [395]).

HIV-1 DNA and mRNA have been found in the renal globular and tubular epithelial cells of kidney, suggesting replication in renal tissues. Kidney biopsies have shown the presence of circularized viral DNA, suggesting active replication [396,397]. Recent studies showed increased evidence of the kidney as a potential reservoir where the virus is exchanged between interstitial T cells and renal tubule epithelial cells [398]. Using novel noninvasive tests designed for determining HIV-1 infection of the kidney allografts by measuring HIV-1 DNA and RNA levels in a patient’s urine, it has been demonstrated that HIV-1 can be detected in kidney allografts after transplantation despite undetectable viremia, and this infection might influence the graft outcome [399]. Another study suggested that the HIV-1 persistence is reduced in the transplant recipient following the transplantation of the HIV-1 infected kidney [400].

Studies conducted by Ravazzolo *et al.* indicated the presence of SRLV provirus transcripts (both un-spliced and multi-spliced) in alveolar macrophages, confirming that the lungs represent an important site of virus replication [369,401,402]. Other studies also confirmed relatively high levels of genomic and sub-genomic viral RNAs in alveolar macrophages of infected goats [403]. In earlier studies, it was found that SRLV-infected sheep alveolar macrophages were able to stimulate CTL activity *in vitro* and were targets of these activated CTL [404]. In recent studies, the increased expression of chemoattractant chemokines (MCP-1 and IP-10) was linked to the inflamed lungs [50]. Some fatal infections show diffuse interstitial pneumonia characterized histologically by severe lymphoplasmacytic infiltrates with massive alveolar proteinosis, interstitial fibrosis, and type II pneumocyte hyperplasia [405].

## 10. Can SRLV Help to Develop Strategies to Control HIV-1?

SRLVs in infected sheep and goats do not cause immunodeficiency like HIV-1 does in HIV-1-infected humans. The reason why SRLVs did not evolve towards an immunodeficiency-inducing phenotype is not well understood. It has been established that SRLVs replicate only transiently in the periphery and replicate productively in macrophages but not in CD4^+^ T cells in target tissues. Our group has demonstrated that SRLVs have Tat-independent constitutive LTR promoters [60]. We have also shown that SRLV infection in newborn calves results in a total clearance of the virus after four months of virus replication and diffusion in target tissues [115].

Taken together, these results helped to conclude that the constitutive expression of viral proteins by SRLV LTRs could have induced antigen-specific T cell responses that were associated with virus clearance. To test the hypothesis, our group has recently generated a chimeric genome, based on the former vaccine integration and replication-defective ∆4-SHIV_KU2_ by replacing the 5’SIV LTR with that of CAEV (∆4-SHIV-KU2 was derived from SHIV-KU2, from which the *rt*, *int*, *vif*, and the 3’ LTR sequences were deleted). The replication- and integration-deficient lentivector DNA vaccine showed promising results by inducing potent immune responses both in mice and macaques [406].

These encouraging results led to the development of a further enhanced immunogenic chimeric lentivector DNA also based on the SHIV genome in which both 5’ and 3’ LTRs of SIV were replaced with those of CAEV. Additionally, the integrase was deleted, making it a safe, novel, non-integrative one-cycle lentivector DNA vaccine that produces viral antigens and particles to stimulate the immune system in *in vivo* transfected cells. These viral particles can transduce target cells without integrating vaccine DNA into these cells, thereby expressing further viral proteins and thus amplifying the vaccine antigenicity. This lentivector induced high multifunctional persistent immune responses both in immunized mice and macaque models [406]. 

These data provide an encouraging perspective of use of CAEV for the development of innovative strategies for the generation of a safe and long-term effective vaccine that could control HIV-1 [407].

## 11. Conclusions 

HIV-1, which causes AIDS, has been responsible for the infection of over 75 million people and the death of nearly 35 million people worldwide. SRLVs infect goats and sheep in flocks all over the world. The reason why these have not evolved to induce AIDS in their natural hosts in contrast to primate lentiviruses remains not well understood. Knowledge from SRLV studies was very helpful to understanding HIV-1 biopathology and host/pathogen interaction early after the discovery of the human lentivirus. However, during the last decades, there was no substantial effort to use the SRLV model for better understanding the complex host/pathogen interaction of primate lentiviruses. Comparative analyses of structural, functional, and pathogenesis differences between SRLVs and primate subgroups of lentiviruses might bring new insights that will help us better understanding the natural history of these viruses and help develop innovative tools to control them. 
